# Report of Thirteen Operations for Goitre, and Technique Employed

**Published:** 1895-07

**Authors:** B. E. Hadra

**Affiliations:** San Antonio, Texas


For Texas Medical Journal.

### REPORT OF THIRTEEN OPERATIONS FOR GOITER, AND TECHNIQUE EMPLOYED.

BY DR. B. E. HADRA, SAN ANTONIO, TEXAS.

Read before North Texas Medical Association, June, 1895.

I WISH to report briefly my experience in surgery for goiter. It is not so extensive as to make my notes very valuable; but since, to my knowledge, there exists quite a dread of surgical interference, as well among physicians as among patients, my report may serve some purpose.

I well know that, recently, less heroic means, especially injections of iodoform, feeding with thyroid, gland, etc., have proven to be of great value in reducing or removing the new growth; still, there will always be cases where the knife will be the last resort.

I have operated on thirteen cases in the last seven years, all of which, I think, were cured, since I was never informed of a relapse. Fortunately there was not a malignant case among them, and no one required a total extirpation of the gland.

I will, therefore, limit my remarks to the surgery of these simpler and more tractable forms of goiter.

Amongst the thirteen patients, all of whom were females, only four were born in the United States, and one of the four was a negress, whilst the rest were of German or Swiss nativity. Eight goiters were unilateral and four bilateral. Eleven were multiple colloids, and one of them had a sarcomatous look, but, although portions of the diseased tissue were left behind, the tumor totally disappeared. Only one was an uncomplicated cyst. It had a fine wall and contained transparent fluid. It was the size of a large apple, and sprang from the isthmus. It was so bare of blood-vessels that no ligature was needed. One was a semi-solid tumor with a smooth cyst wall, a so-called fœtal adenoma.

Being guided by the directions of Kocher, Woelfler and Socin, I gradually worked out an operation which has given me such full satisfaction, and made it so easy and safe, that I think its description, though there is nothing original in the device, may help others. It meets the most important objects, namely: not to remove all the gland in order to avoid cachexia or mixœdema, and also to avoid injury of the laryngeal nerves. Diseased tissue, that may occasionally be left behind, if not too extensive, will shrink and disappear, as is stated by Wolff, and others, and which I found to be a fact in two or three cases.

My procedure is the following: In an unilateral or medial tumor, a long median incision from the jaw to the sternum is made; in a bilateral case, Kocher's crescentic incision from one sterno-cleido mastoid muscle to the other, the highest convexity close to the sternum, is preferable on account of its least disfiguring scar. Then the platysma is divided in the same line. Next the muscles are separated in the median line, drawn to the sides, and only when necessary, nicked or severed, to be afterwards reunited. Now the most important point to facilitate the operation is to free the tumor of that fine capsule, sometimes containing muscular strata, which binds the organ down. This membrane has to be well divided, and to be broken all over by detaching it from the gland, sideways and downward. Then, and only then, the gland or the tumor can easily be brought, with all its processes, into the field of operation by the operator's fingers. This maneuver never must be omitted, or one will have to work in the depth and in the dark. Now, I take the diseased raised portion of the gland, as much as conveniently can be grasped, at its deepest point, between the index finger and thumb of my left hand, compressing in this way the blood vessels from below, and then the incisions are made in the direction where the large veins can best be avoided. The incisions have to go through the whole affected portion down to your left hand fingers, and have to be repeated until all the nests of colloid accumulations are exposed. They then are well scooped and scraped out with a sharp spoon, and with the compressing fingers still controlling hemorrhage, the remaining walls, which consist of healthy parenchyma (pared, if they seem to be too massive) are brought together with a running catgut suture until they are drawn well together into one mass. This last part is best done by the assistant, so as to let the operator keep his hold on the blood vessels. Of course, this procedure has to be repeated on other portions, if necessary. The wound is then closed with any material the surgeon prefers, and with or without drainage.

I would warn against using silk for the deep sutures. It may not remain dormant but cause fistulous tracts, as has happened to me once, necessitating the re-opening of the wound to remove the foreign body. I would add that almost always a moderate fever followed my operations, which was, no doubt, an absorption fever, and should not cause any alarm.

